# Right to mental integrity and neurotechnologies: implications of the extended mind thesis

**DOI:** 10.1136/jme-2023-109645

**Published:** 2024-02-26

**Authors:** Vera Tesink, Thomas Douglas, Lisa Forsberg, Sjors Ligthart, Gerben Meynen

**Affiliations:** 1 Department of Philosophy, Faculty of Humanities, Vrije Universiteit Amsterdam, Amsterdam, Netherlands; 2 Oxford Uehiro Centre for Practical Ethics, Faculty of Philosophy, University of Oxford, Oxford, UK; 3 Jesus College, University of Oxford, Oxford, UK; 4 Department of Criminal Law, Tilburg University, Tilburg, Netherlands; 5 Willem Pompe Institute for Criminal Law and Criminology and UCALL, Utrecht University, Utrecht, Netherlands

**Keywords:** Ethics

## Abstract

The possibility of neurotechnological interference with our brain and mind raises questions about the moral rights that would protect against the (mis)use of these technologies. One such moral right that has received recent attention is the right to mental integrity. Though the metaphysical boundaries of the mind are a matter of live debate, most defences of this moral right seem to assume an internalist (brain-based) view of the mind. In this article, we will examine what an extended account of the mind might imply for the right to mental integrity and the protection it provides against neurotechnologies. We argue that, on an extended account of the mind, the scope of the right to mental integrity would expand significantly, implying that neurotechnologies would no longer pose a uniquely serious threat to the right. In addition, some neurotechnologies may even be protected by the right to mental integrity, as the technologies would become *part of* the mind. We conclude that adopting an extended account of the mind has significant implications for the right to mental integrity in terms of its protective scope and capacity to protect against neurotechnologies, demonstrating that metaphysical assumptions about the mind play an important role in determining the moral protection provided by the right.

## Introduction

 Neurotechnology is advancing at a rapid pace. Neurotechnological devices that intervene in the brain are capable of inducing a wide range of neuronal, mental and behavioural changes.^1^,^4^ The technologies are becoming more sophisticated and efficient, and appear capable of collecting neural data and manipulating mental states with increasing ease. The access to the mind these neurotechnologies provide has incited debates about the need to protect our minds. As Bublitz writes, ‘the greater the extent to which the skull as the natural barrier of the mind becomes permeable, the more pressing the need to draw normative limits to interventions’ (p. 390).^5^

Accordingly, scholars have started to explore which moral (and legal) rights could protect us against the use and misuse of these technologies.^6^,^9^ While we have relatively well-defined rights to protect our bodies, such as the right to bodily integrity,^10^ some suggest that the mind should be given greater moral protection than can be provided by bodily rights. It is, for instance, claimed that mental properties are ‘significantly different from physical properties of the body and brain’ (p. 111)^7^ and therefore require a different level of protection, and that ‘harm to mind is in many ways different from harm to body’ (p. 56).^11^ This has led to proposals for a separate right to ‘mental integrity’ to protect our minds.^7 8 11^

An ensuing question concerns the scope of such a right to mental integrity. The boundaries of the body are, in general, clear, making identifying interferences with it rather straightforward.^1^ The mind, however, still eludes a clear and agreed definition, and the metaphysical underpinnings of the mind remain a matter of live debate in the philosophy of mind.^12^ In defining a right that protects the mind, it seems necessary to rely on at least *some* metaphysical ideas about the mind to delineate *what* is to be protected. As noted by Bublitz and Merkel, for matters involving the protection of the mind, ‘there is no ‘ethics without metaphysics’’ (p. 54).^11^ Nevertheless, in the ethical literature on the right to mental integrity, discussions of the metaphysical boundaries of the mind, even though seemingly crucial to defining the protective parameters of a right that is supposed to protect it, are often lacking.

Instead, (implicit) assumptions about the boundaries of the mind are often made. As we interpret them, most of the proposals for a right to mental integrity implicitly assume an internalist view of the mind.^2^ This view holds that our mind is in some sense (where the precise sense differs across different versions of internalism) ‘contained within’ the body and, on many versions of internalism, the brain more specifically. Internalism is arguably the most widely assumed view, including among philosophers of mind,^13^ and is often adopted without perceived need for defence by scholars outside of the philosophy of mind.^14^ However, in recent years, *externalist* views—according to which our minds are not wholly contained within our brains or bodies, for example, because they also crucially rely on or are realised by external artefacts and processes—have gained popularity.^15^,^3^ This trend appears to be at least partly motivated by neurotechnological advancements, which increasingly seem to blur the boundaries between our biological brains and artefacts external to the brain.^16^ Think, for instance, of the development of brain-computer interfaces (BCIs) that can establish bidirectional and real-time connections between brains and computers to restore or augment mental capacities.^17^ These rapidly developing technologies are increasingly capable of performing functions that were traditionally performed by the brain, and for some, this casts doubt on whether a sharp distinction between the internal and external (to the brain) should be upheld in defining the boundaries of the mind.

Assuming an internalist view when defending the right to mental integrity ensures that the right protects what is within the skull. However, as long as the question of what exactly constitutes our minds remains an open one, we might also consider what adopting different—externalist—views of the mind would imply for the right to mental integrity and its protective scope.

In this article, we explore the implications of one such externalist view—the ‘extended mind’ thesis—for the right to mental integrity.^4^ To clarify, we do not argue for adopting the extended mind thesis (EMT); we merely consider the implications that adopting such a view would have. First, we consider how the right to mental integrity is standardly understood in the literature. Second, we outline the EMT and consider its appeal. Third, we discuss two implications of adopting an extended mind view for the right to mental integrity and its capacity to protect against neurotechnologies, that is: (1) the scope of the right will expand significantly and neurotechnologies are therefore no longer unique among interventions in the seriousness of the threat they pose to mental integrity, and (2) neurotechnologies may fall under the protection of the right to mental integrity themselves.

## The Right to Mental Integrity

The right to mental integrity is less well established than the right to bodily integrity. Yet, in the law, a legal right to mental integrity has gained some acceptance.^22^ For instance, mental integrity is protected by Article 9 of the International Covenant on Civil and Political Rights.^23^ A legal right to mental integrity is also explicitly guaranteed by Article 3 of the European Charter of Fundamental Rights, which states that ‘everyone has the right to respect for his or her physical and mental integrity’. Similarly, Article 17 of the Convention on the Rights of Persons with Disabilities guarantees the protection of ‘physical and mental integrity,’ and Article 8 of the European Convention on Human Rights asserts both a right to bodily and ‘psychological’ integrity. However, these sources give no account of what constitutes mental integrity, and as noted by the United Nations Special Rapporteur on Freedom of Religion or Belief, the scope of the right has also not been clearly defined in case law.^24^

If we turn to legal scholarship, we find more elaborate characterisations of the right. In their 2014 paper, Bublitz and Merkel make a case for explicitly recognising the right to mental integrity, which they take to be already implicit in law.^11^^5^ This right, as they understand it, ‘protects freedom from severe interferences by the state and third parties, setting up a defensive wall against unwanted intrusions through both factual interventions and normative obligations’ (p. 58).^11^^6^ As examples of interferences that could infringe this right, they mention subliminal imagery, the spiking of drinks with brain-active drugs and non-consensual transcranial magnetic stimulation. Ienca and Andorno have similarly called for the introduction of a right to mental integrity into human rights frameworks, since doing so, in their view, is necessary to adequately address emerging threats to our minds posed by novel neurotechnologies.^25^ They understand the right to mental integrity as ‘the right of all individuals to protect their mental dimension from potential harm’ (p. 17).^25^ They contend that this right ‘should provide a specific normative protection from potential neurotechnology-enabled interventions involving the unauthorized alteration of a person’s neural computation and potentially resulting in direct harm to the victim’ (p. 18).^25^

It is unclear whether these authors regard the legal right to mental integrity as enforcing a moral right to the same. However, several philosophers have defended a moral right to mental integrity. For instance, Craig posits a right that ‘protects the inner-sphere of the human person’ (p. 116).^7^ Another proposal for recognition of a moral right to mental integrity comes from Lavazza, who defines mental integrity as ‘the individual’s mastery of his mental states and his brain data’ and suggests that the right protects an individual from having their mental states and brain data read or modified without valid consent.^8^ In a subsequent article, Inglese and Lavazza again stress the importance of protecting mental integrity, which they define as ‘the ability to formulate thoughts, judgments and intentions, make plans and implement them without direct external interference of any kind due to neurotechnology’ (p. 2).^26^

When it comes to neurotechnologies and moral (and legal) rights, criminal justice is often where the rubber hits the road, as in this societal domain, far-reaching measures—some of which are intended to have mental effects—may be taken without the recipient’s consent. Therefore, it is not surprising that the right to mental integrity has been invoked in relation to the potential use of neurotechnologies in criminal justice contexts, such as in a forensic psychiatric setting. For instance, Shaw stresses the importance of the right in protecting criminal offenders from mandatory neurotechnological interventions. She characterises the right as protecting individuals from non-consensual interference with the mind^27^ and proposes that it can be infringed by ‘intentionally interfering with a person’s mental states through non-rational means’ (p. 1418).^28^ Similarly, discussing the right in the context of criminal justice, Birks and Buyx argue that we can interfere with an individual’s mental integrity when we ‘intentionally create or alter one of his desires through means other than engaging with that person’s autonomous thought’ (p. 136).^29^

While the different accounts just surveyed differ in how they understand the moral right to mental integrity (henceforth, just ‘the right to mental integrity’), the general consensus seems to be that it is a right that, perhaps among other things, protects against non-consensual mental interference, or certain forms thereof.^7^

Many of the abovementioned proposals for a right to mental integrity have arisen in response to emerging neurotechnologies. These are believed to be capable of interfering with our minds in unprecedented ways, and the directness and ease with which neurotechnologies can target mental states are often thought to make them unique in the seriousness of their threat to the mind. While Bublitz and Merkel’s proposal for a legal right to mental integrity also refers to, for instance, using subliminal imagery—an intervention that does not directly target the brain—as potential interference with mental integrity,^11^ many other proposals refer specifically to emerging neurotechnology as the prime motive for introducing or recognising the right.^30^^8^

Implicit in the proposals for introducing or recognising a right to mental integrity as a response to *neuro*technologies appears to be the internalist assumption that the mind is contained within the brain. While some of the proposals include a brief discussion of the metaphysics of the mind, such as the one by Bublitz and Merkel,^11^^9^ most of the abovementioned literature on the right to mental integrity does not explicitly engage in metaphysical discussions of what constitutes the mind but instead seems to implicitly endorse an internalist view. For if an externalist view were assumed and the mind were thought to rely on or be realised in external cognitive aids as well, we might have expected that developments in, for example, information technology would also have motivated the adoption or recognition of a right to mental integrity, or at least would have been widely invoked as potential threats to the right. However, whether the mind is contained within the brain remains a matter of lively debate in the philosophy of mind^31^ and the internalist view therefore a mere assumption.

Interestingly, the same advances in (neuro)technology that have motivated the proposals for a right to mental integrity have also prompted discussions regarding the possible extension of the mind beyond the brain,^16 32^ as neurotechnologies progressively blur the lines between human and machine. Think, for instance, of closed-loop deep brain stimulation (DBS), capable of reading brain activity and ‘autonomously’ intervening to change mental states or behaviour,^33^ or neuroprostheses that can replace impaired or missing brain functions such as vision.^34^ BCIs can establish bidirectional communication links between the brain and external devices and are already capable of restoring speech and motor functions.^35 36^ The capacity of technological systems to perform functions traditionally performed by biological systems renders it increasingly plausible for some that the mind can also be partly realised by physical entities external to the brain.

## The extended mind thesis

One view on which the mind is not confined within the brain, but actually extends beyond it, is the EMT.^37^ As we will understand it, the EMT holds that mental states that make up the human mind—including beliefs, desires and memories—are not only realised by our brains but can also be realised by physical processes and artefacts located outside the brain and indeed beyond the body.^38^^10^

The thesis can best be explained using the paradigmatic example of Inga and Otto provided by Clark and Chalmers.^38^ Inga hears of a new exhibition at the Museum of Modern Art. She recalls that the museum is located on 53rd Street and leaves her house to visit the museum. Otto also hears of the new exhibition at the Museum of Modern Art, but he suffers from dementia and has a poor memory. He uses a notebook to write down new information he learns and to look up old information he wants to recall. Because Otto wants to visit the exhibition, he consults his notebook and sees that the museum is on 53rd Street, and so he walks to 53rd Street to the museum. Clark and Chalmers argue that Otto’s notebook plays the same role in Otto’s memory as the memory-encoding neurons play in Inga’s memory. Therefore, they argue, we should consider Otto’s notebook to be *constitutive* of his mind. Clark and Chalmers introduced the *parity principle* to establish whether or not an external artefact such as the notebook should be considered part of the mind. This principle holds that if something in the external world plays such a role in a mental process that we would have no difficulty counting it as part of a mental process if it were internal, it should be counted as part of the mental process—regardless of whether it is internal.^38^

Defenders of the EMT hold that people with impaired cognitive performance such as Otto are not alone in having extended minds, but rather that almost every human being does. For instance, humans often engage in so-called cognitive offloading, which involves using external artefacts as a cognitive ‘aid’ to free up internal resources for other tasks. Think, for instance, of writing down a mathematics problem that is (for most) impossible to solve without external aids, such as 854×362. When someone solves such a problem, pen and paper are used to decompose the complex task into simpler tasks which are then solved—a process that integrates seamlessly internal (ie, biological) computing with external computing. Another example is the use of smartphones, which many of us (continuously) use for a variety of tasks that would be construed as mental abilities if they were internal.

An important point of discussion regarding the EMT is the degree of *integration* between internal and external that is necessary for an external artefact to count as part of the mind. As we saw, Clark and Chalmers endorse the parity principle. However, some have argued for more demanding integration conditions, on the basis that the parity principle would lead to ‘cognitive bloat’.^39^ For instance, Palermos argues that what is required is tight coupling in the form of ongoing feedback loops—a principle based on dynamical systems theory.^40^ It implies that two systems are coupled and form an integrated system when they bidirectionally influence each other continuously when performing a task, which would be a prerequisite for the mind to extend to the coupled system. This would, for instance, prevent artefacts such as a camera used to take one picture from becoming part of the mind and only allow for artefacts that we *bidirectionally* and *continuously* interact with (eg, smartphones or laptops) to become external physical constituents of the mind. Although much more has been said about the conditions for integration required for mind extension, we will not further outline this discussion here. Instead, we will restrict our analysis to artefacts and technologies—such as smartphones and neurotechnologies—that our minds seemingly most intimately interact with and would therefore likely satisfy many of the integration conditions that have been proposed in the literature.

When first introduced, the EMT was considered rather radical, but it has been gaining popularity in recent years.^41 42^ Although it would be an exaggeration to describe the EMT as a dominant view,^11^ even those not convinced that our minds are currently partly realised by external objects may accept that with further technological development, they could be in the future. Most of us rely on technology from the moment we wake up until the moment we go to bed.^41^ We use an alarm clock to wake us up, use the global positioning system on our smartphone to navigate through the city, use a computer for many (if not most) work-related tasks and let our smartphones suggest a movie to watch before we go to bed. This constant cognitive offloading due to technological developments has increasingly ‘normalised’ the high dependence on external artefacts for performing cognitive tasks.^43^ In the future, technologies may evolve to such an extent that they become more intimate, integrated and powerful—and more closely resemble what we would consider ‘normal’ mental processing.^16^ These developments could well increase the plausibility of the EMT as a thesis that questions the relevance of a sharp distinction between the biological and the technological in defining the mind and mental processing.

## Implications of the extended mind thesis for the right to mental integrity

If internalist assumptions were to be discarded and instead the EMT were to be adopted, this would have, as we will argue below, significant implications for the right to mental integrity and its capacity to protect against neurotechnologies. More specifically, we argue that (1) the scope of the right would expand significantly and neurotechnologies would not constitute the unique threat to mental integrity they are often assumed to be, and that (2) neurotechnologies might fall within the protective scope of the right themselves.

### The scope of the right to mental integrity expands

If we discard the conception of mental states as exclusively realised by the brain and allow for mental states to be (partly) realised by artefacts beyond the brain and indeed body, the protective scope of the right to mental integrity would expand significantly.

As stated above, the right to mental integrity is generally understood as a right that protects against non-consensual mental interferences. There are different views on what counts as mental interference, but, following Bublitz and Merkel,^11^ we believe that it should be uncontroversial that this category includes at least the intentional alteration of a person’s mental states by physically altering the physical basis of those mental states.^12^ On ‘traditional’ internalist accounts, this would imply alterations to *brain* states. On the EMT,^13^ this could *also* include alterations to external artefacts that can be constitutive of mental states.

Let us return to the example of Inga and Otto. If someone were to go through Otto’s notebook and rip out the page containing the location of the museum right after Otto mentioned he wanted to visit it, this could, on the EMT, infringe his right to mental integrity. For, on the EMT, this intervention could count as physically altering the physical basis of some of Otto’s mental states, just as would neurochemically altering the brain circuits implicated in Inga’s memory retrieval. The difference lies merely in whether the physical realiser that is interfered with is inside the brain or outside the brain and the body. Consequently, we must accept that if we would consider interference with Inga’s brain that rearranged her memories an infringement of her mental integrity, we must also consider the interference with Otto’s notebook to infringe his mental integrity, assuming that the intentions are the same in both cases and that the notebook is indeed part of the external basis of Otto’s mind. Clark and Chalmers already noted that ‘in some cases interfering with someone’s environment will have the same moral significance as interfering with their person’ (p. 18),^38^ and others have also emphasised this moral implication of accepting the EMT.^14 18 21 32 46^

It is important to stress that not all external artefacts that a person uses would fall within the scope of the right to mental integrity according to the EMT—only those that satisfy the conditions for being physical realisers of mental states would do so. As mentioned above, there is no consensus about these conditions, but regardless of the exact criteria, if external entities—however many—can be physical realisers of the mind, the scope of the right will expand to include them.

Similar arguments have been developed by legal scholars regarding several legal rights. For instance, Blitz applies the EMT to the right to freedom of thought and argues that the right should not only protect our ‘natural’ ability to engage in reflection, but ‘it should also lead courts to identify and protect technologies and resources that support mental autonomy and externalised thought’ (p. 35).^47^ Carter and Palermos consider the EMT in the context of personal assault and argue that legal theory and practices will need to expand their conception of personal assault so that it includes intentional harm to external artefacts that are sufficiently integrated with our mental machinery.^43^ Palermos contends that an extended mind perspective would have significant implications for the right to mental privacy, as it would imply that information stored in external devices, which is relatively easy to access, could include mental data.^20^ He therefore argues for expansion of the right to mental privacy to include such data if the EMT holds true.

This expansion of the protective scope of the right to mental integrity on the EMT suggests that neurotechnologies do not pose the uniquely serious threat to the right they are—on an internalist account—generally assumed to pose. It is often argued that physical interventions into the brain are, other things being equal, more morally problematic than other ways of altering a person’s mental states (eg, via cognitive therapy).^8 11^ Underlying such claims is a sharp moral distinction between the internal and the external, where intervening in the external environment is considered to raise fewer moral worries than intervening in the internal physical realisers of mental states.^14^ Because the EMT rejects such a distinction, according to the thesis, a considerable number of interventions *besides* interventions into the brain can alter physical realisers of mental states and thus can also just as easily and just as directly infringe the right to mental integrity.^14^ Therefore, it seems that accepting the EMT forces us to accept that interventions such as tampering with someone’s smartphone can infringe the right to mental integrity in the same way as tampering with their brain states can—and thus that neurotechnologies do not pose a more serious threat to mental integrity than external interventions.^48^^15^ (Of course, there may be other reasons—independent of the right to mental integrity—for assuming that neurointerventions are more morally problematic than other interventions on external realisers of mental states. For example, interventions on internal realisers may also infringe the right to *bodily* integrity.^10^)

### The right to mental integrity may protect neurotechnologies

Accepting the EMT means accepting that artefacts external to the brain can also be partly constitutive of mental states. Neurotechnologies, especially those that are permanently implanted in the brain, would seem prime candidates for such non-neural mind constitution. Devices such as DBS or BCIs arguably have the potential to become more integrated with mental processes than any other external artefact. They are in close physical proximity to the brain, could be in continuous intimate interaction with (other) mental processes and can play a significant role in the mental functioning of a person. If such technologies will indeed be part of a subject’s mind, it would seem that these neurotechnologies themselves could be protected by the right to mental integrity.

Consider the following case of closed-loop neuromodulation in a patient with depression as described by Scangos and colleagues.^49^ The patient had childhood-onset depression and was unresponsive to multiple antidepressant treatments. A DBS device was implanted in her brain, with electrodes placed in brain regions that were identified with electrophysiological methods to be associated with her depressive symptoms. The DBS acted in a closed loop, as the device would ‘read’ brain activity and stimulate these regions only when their activity reached a certain threshold—signalling the onset of certain symptoms. The stimulation by the DBS device resulted in significant and sustained symptom improvement, with the patient experiencing reduced depressive symptoms and anxiety, and stimulation in certain areas led the patient to experience pleasurable and energising states.

The implantation of the DBS device in the patient could be conceived of as mental interference, as the electrical currents alter the patient’s mental states by intervening in the internal physical realisers of those states. However, if we were to accept the EMT, it might be argued that, at least after some time, the DBS device becomes one of the physical realisers of the patient’s mental states. The device continuously interacts with her neural activity—it ‘reads’ brain activity and ‘writes to’ the brain in response to this activity, that is, when a certain symptom is signalled to arise, to alter the patient’s mental states. In light of this intimate feedback loop between the DBS device and the patient’s mental states, we may assume that the device fulfils a set of strict integration requirements such as those proposed by Palermos^40^ and thus becomes a physical realiser of the patient’s mind on the EMT. In such cases, where brain devices become external physical realisers of the person’s mind, they would plausibly also fall within the scope of what the right to mental integrity protects—and could thus become subject to infringements of the right themselves.

To illustrate this point further, let us consider a more recent case described by Gilbert and colleagues.^50^ Patient R was implanted with a BCI designed to detect epileptic activity in the brain and alert the patient to the onset of seizures. The BCI device had considerable effects on Patient R’s mental functioning, as she described experiencing ‘de novo agential capacities which appeared inseparable from functioning with her implanted device’ (p. 784).^50^ After wearing the device for several years, the manufacturer of the device forced Patient R to take out the BCI device due to financial constraints. As a result, Patient R experienced ‘radical psychological discontinuation and disruption of agential capacities, which continue to cause persistent emotional and affective harms years after system operator removal’ (p. 784).^50^

On an internalist view, the removal of the device might seem to ‘release’ Patient R from continuous (though consensual) interference with her mental states. However, if the EMT holds true, the removal of the device might actually constitute an infringement of her right to mental integrity. While it is still the case that the electrical currents altered the physical realisers of Patient R’s mental states, the device itself is now *also* one of the physical realisers of her mental states. If the device were removed, one could argue that a part of R’s mind would be removed—which would plausibly interfere with her mental integrity. The external artefact has now become part of what constitutes her mind and thus what is protected by the right to mental integrity. In other words, on the EMT, the right to mental integrity might also *protect* neurotechnologies rather than only protect *against* them.

## Further moral implications

The assumed boundaries of the mind determine the parameters of the moral protection provided by the right to mental integrity and will thus bear on what kind of interventions we think can permissibly be used in light of this right. To illustrate how adopting an alternative metaphysical view such as the EMT might lead to different moral assessments, let us turn to the current debate on the use of neurotechnologies in the context of criminal justice. There has been some discussion over the past years about the possibility of using neurotechnologies on criminal offenders to facilitate rehabilitation, potentially in exchange for reduction or removal of a prison sentence.^951^,^53^ The right to mental integrity has been invoked in discussions regarding the ethical permissibility of using neurotechnologies in this context.

More specifically, an often-presented argument *against* the use of neurotechnologies in this context is their potential to infringe the right to mental integrity.^7 27 29^ Neurotechnologies may alter the mental states of offenders in significant ways to make them change their behaviour. Incarceration might also interfere with (or at least influence) mental states. However, it does so less directly and is therefore considered less of a threat to mental integrity.^29^ These ethical evaluations thus seem to imply that neurotechnologies are more problematic than incarceration with respect to the right to mental integrity.^16^^17^ However, as we argue below, such an argument seems less plausible if we accept the EMT.

If the EMT holds true, incarceration could infringe the right to mental integrity in the same way that neurotechnological interventions do. After all, imprisonment involves removing someone from their familiar social, professional, intimate and technological environment, which arguably involves separating them from many external artefacts that have become partly constitutive of their mental states (eg, their smartphones). By placing a convicted offender in a severely impoverished environment (prison), the offender may lose a considerable part of his mind through physical interference with its (external) physical basis. Hence, on the EMT, incarceration could be just as morally problematic as neurotechnology use with regard to the right to mental integrity (depending, of course, on the further impact both would have on the person—this may be relevant to the seriousness of the infringement).^18^

One could argue that, also on internalist accounts, creating an impoverished environment through incarceration might infringe the right to mental integrity because of its reported negative effects on mental states of offenders.^54^,^56^ However, on internalist accounts, it is by virtue of such ‘secondary’ mental effects that incarceration might infringe the right. On the EMT, on the other hand, incarceration may infringe offenders’ right to mental integrity *regardless* of whether they experience any further negative mental effects; incarceration entails serious deprivation which arguably infringes the right to mental integrity just by virtue of directly interfering with part of its (extended) physical basis.

This is also a relevant factor in how we might assess the ethical permissibility of neurotechnologies, for instance, the discontinuation of their use. Consider again the case of Patient R.^50^ After the manufacturer forced her to remove the device, Patient R experienced ‘radical psychological discontinuation and disruption of agential capacities, which continue to cause persistent emotional and affective harms years after system operator removal’ (p. 784).^50^ Whether or not we accept the EMT, the removal of the BCI might constitute an infringement of the patient’s right to mental integrity. On an internalist account, the removal of the device might infringe the right because of the mental anguish Patient R experienced. On the EMT, however, the removal of the device plausibly *itself* infringes the right because it involves the removal of a physical realiser of her mind. The further mental effects R experiences are less relevant to establishing the infringement (although they may add to its seriousness). This means that the removal of neurotechnologies could, in some cases, more readily be considered ethically impermissible on the EMT because of such inherent mental interference.

Accepting the EMT thus has at least two important implications for how the right to mental integrity bears on neurotechnologies. First, it implies that neurotechnologies are not uniquely serious in the threat that they pose to mental integrity. It may be that neurotechnologies can constitute especially serious or clear infringements of the right by virtue of acting physically on the physical basis of the brain. However, if the EMT holds, neurotechnologies are not alone in having this feature; environmental interventions that act on the external physical realisers of the mind have it too. Second, since neurotechnological devices can themselves be among the external realisers of the mind, it follows that that they might be protected by the right to mental integrity themselves. Altering, tampering with or terminating neurotechnological devices, even those that are external to the brain, can itself threaten mental integrity in a similarly direct way as can the implantation or activation of these devices. Also, from a procedural perspective, on the EMT, there might be less need to ‘prove’ that interventions such as incarceration or the discontinuation of a neurotechnology actually induced mental changes, as they will already infringe the right to mental integrity by directly interfering with externally realised mental states.

## Conclusion

The right to mental integrity is taken to provide protection against neurotechnologies, but the extent of the protection provided by the right is contingent on metaphysical assumptions regarding the mind that are rarely explicated in the ethical literature. If we discard the commonly endorsed internalist view of the mind as brain-based and, instead, adopt the view that it can extend beyond the brain and indeed the body, this has serious implications for the right to mental integrity and the extent of its protection. On the extended mind thesis, the scope of the right to mental integrity would expand significantly, and it could be infringed as directly and easily by environmental interventions as it is by neurotechnological ones. Moreover, external artefacts such as neurotechnologies could also become physical realisers of the mind and therefore themselves receive protection from the right to mental integrity. The assumed boundaries of the mind appear to play a crucial role in defining mental rights and their protective scope.

## Data Availability

No data are available.

## Footnotes

The boundaries of the body in the context of a right to bodily integrity are, however, not entirely uncontested. For discussion, see, for instance, Bublitz,Aas,^58^ AasBublitz^57^ and Tesink *et al*..^10^

This claim will be defended later in the paper.

Note that ‘internal’ and ‘external’ traditionally denote internal and external to the body, as separated by the boundary of the skin. However, within the philosophy of mind, internalism—in its most common form—assumes a specific part of the body, namely, the brain, to be the crucial determinant of the mind, and is therefore here more often understood as ‘internal to the skull’. This is also illustrated by the fact that an embodied cognition view, a view that strictly speaking defines the mind in terms of what is internal to the boundaries of the skin, is often labelled an externalist position and even ‘anti-internalist’.^59^ When making the distinction between internal and external throughout our analysis, we use ‘internal’ to refer to the brain and ‘external’ to everything outside the brain.

Several authors have explored the implications of the extended mind thesis for rights over, or duties with respect to, the mind. See, for example, Levy,^14^ Anderson,and.^32^ Buller,^18^ Glannon,^19^ Palermos^20^ and Clowes *et al.*^21^ Our discussion differs from these earlier discussions in two regards. First, we are focusing specifically on *one* putative right of the mind: the right to mental integrity. Second, our aim is not to explore the ethical implications of the extended mind thesis as such,, but rather to challenge the commonly (and often implicitly) adopted internalist assumptions in proposals for a right to mental integrity by demonstrating what adopting non-internalist assumptions would imply for its protective scope. We use the extended mind thesis as an exemplary case of non-internalist metaphysical assumptions about the mind in light of its relative radicality.

Bublitz and Merkel refer primarily to criminal and human rights law.

While they refer to it as a right to ‘mental self-determination’, in later articles, Bublitz has also referred to a right to ‘mental integrity’ to indicate the same.^5 60^

This characterizationcharacterisation of the right is schematic and would need to be more fully specified—for example, by being supplemented with an account of mental interference. Such accounts are scarce in the literature, but gaining more attention (see, e.g.eg, Zohny *et al.*,^61^). Moreover, as the ‘perhaps among other things’ indicates, we acknowledge that the account may be incomplete in the sense that it does not capture the right’s full scope. For example, the right may also include positive elements such as a right *to* certain forms of control over one’s own mind. However, the schematic and possible incomplete account offered here will suffice for our purposes in this paper. In addition, note that we do not make a distinction between interference with occurrent and non-occurrent mental states, as we will assume both to have the potential to interfere with mental integrity.

A reference to (neuro)technology has been identified by Douglas and Forsberg as one of the three most frequently used rationales to justify the need for a right to mental integrity.^30^ While they refer to rationales for a *legal* right to mental integrity, the arguments they identify could also be applied to a moral right to mental integrity, although they do not explore this further in the article.^30^

Bublitz and Merkel^11^ suggest in their proposal for a legal right to mental integrity that the law should adopt the most ”innocuous“ position which they believe is a weak supervenience view, implying that “there can be no changes on the mental level without some change on the physical, that is, neuronal level”[11, (p. 54]).^11^ While a supervenience view can be compatible with externalist theories, their reference to only the ‘neuronal level’ as supervenience base for the mental seems to imply an internalist view.

As mentioned before, the EMT is only one externalist account of the mind. Other forms of externalism are so-called *content* externalist views, which argue that the content of mental states is not solely determined by neural activity. These include embedded views, which argue that that mental states are causally scaffolded by external artifactsartefacts and practices, and enacted views, which adopt principles of dynamical systems theory to define mental states as arising through dynamic and intimate interaction between the subject and its environment.^15^ The extended view is the most ‘extreme’ externalist view as it endorses *vehicle* externalism, claiming that mental states can actually be *realized*realised by external artifactsartefacts (rather than being ‘merely’ causally dependent on them).

It has many critics,; see, for instance, Rupert.^62^ and Weiskopf.^44^

The physical basis of mental states scancan also be altered by non-physical means, such as psychological means (eg, convincing someone by giving arguments, nudging someone into a different decision, or employing hypnosis). It is clear that not all such interventions infringe the RMIright to mental integrity, but it is a matter of debate whether any do so (see, e.g.eg, Bublitz,^63^ and Focquaert & Schermer^45^).. Levy,,^64^ for instance, argues that also psychological interventions could mentally interfere in a morally problematic way because he argues that only the *effects* of interventions are morally relevant. In light of the ongoing debate on this topic, we restrict our analysis to physical interventions of physical bases of mental states, which we assume to represent the clearest (and least controversial) instances of infringements of the right.

Note that when we use language such as ‘on the EMT’, and we are assuming the truth of the EMT, we do not wish to imply that accepting the EMT has implications for *what* mental rights we enjoy, but we merely consider what accepting the EMT implies for *what is protected* by the mental rights we (seemingly) enjoy such as the right to mental integrity.

Some have disputed this and argued that bodily boundaries remain (most) morally relevant, even if the mind would extend beyond these boundaries. This view has, for instance, been defended by Buller,^18^ who contends that while the mind can be extended, the morally relevant notion of the ‘person’ remains related to the sensory and somatosensory aspects of the body to such an extent that bodily boundaries continue to be ethically relevant.

This reasoning is based on accepting the ‘ethical parity principle’ as articulated by Levy, which in its strong version states that “‘since the mind extends into the external environment, alterations of external props used for thinking are (ceteris paribus) ethically on par with alterations of the brain’.^48^ The soundness of this principle, however, has been contested by some., Forfor instance, by DeMarco and Ford, who argue that there are morally relevant differences between the internal and external—such as fungibility of the external and the locus of consciousness in the internal—that undermine the presumed moral parity between the two.^65^ Such criticisms are generally grounded in a critique of the EMT as a theory, and since we are assuming the accuracy of the EMT for this part of our analysis, we will not further entertain these criticisms.

Within this debate, there has been discussion about a difference in effects on mental integrity between incarceration and neurotechnologies in terms of *intention*. Some have argued that neurotechnologies are less ethically acceptable than incarceration because their effect on mental integrity is *intended*, while the effect of incarceration on mental integrity is merely a *side effect*.^29^ Others have disputed this by claiming that effects on incarceration on mental integrity must also be considered intended, implying that neurotechnologies and incarceration should be treated ethically on par with regards to their effect on mental integrity.^66^ Since this debate does not, we think, significantly add to our discussion, we will not entertain it further.

Not all in the debate agree that there is a morally relevant difference between the mental effects of neurotechnologies and incarceration that would translate to a more serious infringement of the right to mental integrity by the former. For instance, some authors^9 67 68^ question whether the mental effects by incarceration would not also signify serious infringements of the right to mental integrity on certain interpretations of the right as defined by Bublitz and Merkel^11^ or Ienca and Andorno.^25^ We will, however, for the current purposes onlyentertain only the more often-presented argument that neurotechnologies can infringe on the right to mental integrity in ways, or to a degree, that incarceration does not. See, for instance, Craig^7^ and Shaw.^27^

Note that even if one accepts the EMT, one could still argue that using neurotechnologies would be less ethically acceptable than incarceration by appealing to other rights such as the right to bodily integrity.^10^
